# Posterior Averaging Information Criterion

**DOI:** 10.3390/e25030468

**Published:** 2023-03-07

**Authors:** Shouhao Zhou

**Affiliations:** Division of Biostatistics and Bioinformatics, Department of Public Health Sciences, Pennsylvania State University, Hershey, PA 17033, USA; shouhao.zhou@psu.edu

**Keywords:** Bayesian modeling, expected out-of-sample likelihood, Kullback–Leibler divergence, misspecified model, predictive model selection

## Abstract

We propose a new model selection method, named the posterior averaging information criterion, for Bayesian model assessment to minimize the risk of predicting independent future observations. The theoretical foundation is built on the Kullback–Leibler divergence to quantify the similarity between the proposed candidate model and the underlying true model. From a Bayesian perspective, our method evaluates the candidate models over the entire posterior distribution in terms of predicting a future independent observation. Without assuming that the true distribution is contained in the candidate models, the new criterion is developed by correcting the asymptotic bias of the posterior mean of the in-sample log-likelihood against out-of-sample log-likelihood, and can be generally applied even for Bayesian models with degenerate non-informative priors. Simulations in both normal and binomial settings demonstrate superior small sample performance.

## 1. Introduction

Model selection plays a key role in statistical modeling and machine learning. Information theoretic criteria, such as Akaike information criterion (AIC) [1] minimum description length [2] and Schwarz information criterion [3], have been frequently and widely exploited with profound impact on many research fields.

Among these popular methods, a substantial group of model selection criteria was proposed based on the Kullback–Leibler (K-L) information divergence [4]. In the context of model selection, it provides an objective measure to quantify the overall closeness of a probability distribution (the candidate model) and the underlying true model. On both theoretical and applied fronts, K-L based information criteria have drawn a huge amount of attention, and a rich body of literature now exists for both frequentist and Bayesian modeling.

Here we will focus on predictive model selection. To choose a proper criterion for a statistical data analysis project, it is essential to distinguish the ultimate goal of modeling. Geisser & Eddy [5] challenged researchers with two fundamental questions that should be asked in advance of any procedure conducted for model selection:

- Which of the models best explains a given set of data?
- Which of the models yields the best predictions for future observations from the same process that generated the given set of data?

The first question, which concerns the accuracy of the model in describing the current data, has been an empirical problem for many years. It represents the explanatory perspective. The second question, which represents the predictive perspective, concerns the accuracy of the model in predicting future data, having drawn substantial attention in recent decades. If an infinitely large quantity of data is available, the predictive perspective and the explanatory perspective may converge. However, with a limited number of observations we encounter in practice, predictive model selection methods should achieve an optimal balance between goodness of fit and parsimony, for example, as we have seen in AIC.

Compared with frequentist methods, Bayesian approaches to statistical inference have unique concerns regarding the interpretation of parameters and models. However, many earlier Bayesian K-L information criteria, such as Deviance Information Criterion (DIC) [6], follow essentially the frequentist philosophy insofar as they select a model using plug-in estimators of the parameters. Subsequently, the parameter uncertainty is largely ignored. Such a paradigm has changed since the Bayesian predictive information criterion (BPIC) [7], as model selection criteria were developed over the entire posterior distribution. Nevertheless, BPIC has its own limitations, particularly with asymmetric posterior distributions. More importantly, BPIC is undefined under improper prior distributions, which limits its use in practice. More details can be found in Section 3 with a review of alternative methods.

The rest of this article is organized as follows. To explain the motivation of the proposed Bayesian criterion, in Section 2 we review the K-L divergence, its application and development in frequentist statistics, and the adaption to Bayesian modeling based on plug-in parameter estimation. In Section 3, major attention is given to the K-L based predictive criterion for models evaluated by averaging over the posterior distributions of parameters. To select models with better predictive performance, a generally applicable method, named the posterior averaging information criterion (PAIC), is proposed for comparing different Bayesian statistical models under mild regularity conditions. The new criterion is developed by correcting the asymptotic bias of using the posterior mean of the log-likelihood as an estimator of its expected log-likelihood, and we prove that the asymptotic property holds even though the candidate models are misspecified. In Section 4, we present some numerical studies in both normal and binomial cases to investigate its performance with small sample sizes. We also provide a real data variable selection example in Section 5 to exhibit possible differences between the explanatory and predictive approaches. We conclude with a few summary remarks and discussions in Section 6.

## 2. Kullback–Leibler Divergence and Model Selection

Kullback & Leibler [4] derived an information measure to assess the dissimilarity between any two models. If we assume that f(y) and g(y), respectively, represent the probability density distributions of the ‘true model’ and the ‘approximate model’ on the same measurable space, the K-L divergence is defined by

(1) $$KL\left(f||g\right)=\int f\left(y\right)\cdot \log\frac{f(y)}{g(y)}dy=E_{y}\left[\log f\left(y\right)\right]-E_{y}\left[\log g\left(y\right)\right],$$

which is always non-negative, reaching the minimum value of 0 when *f* is the same as *g* almost surely. It is interpreted as the ‘information’ loss when *g* is used to approximate *f*. Namely, the smaller the value of KL(f||g), the closer we consider the model *g* to be to the true distribution.

Only the second term of KL(f||g) in (1) is relevant in practice to compare different possible models *g* without full knowledge of the true distribution. This is because the first term, $E_{y}\left[\log f\left(y\right)\right]$, is a constant that depends on only the unknown true distribution *f*, and can be neglected in model comparison for given data.

Let $y=(y_{1},y_{2},\cdots ,y_{n})$ be *n* independent observations of the data following probability density function f(y). $\tilde{y}$ is a future independent observation following the same density function f(y), representing an unknown but potentially observable quantity [8]. Without exactly knowing f(y), we denote a model *m* with density $g_{m}\left(y|\theta^{m}\right)$ among a list of potential operating models m=1,2,⋯,M. For notational purposes, we ignore the model index *m* when there is no ambiguity. The true model *f* is referred to as the *unknown* data generating mechanism, not necessarily to be encompassed in any approximate model family of $g_{m}$.

As the sample size n→∞, the average of the log-likelihood

$$\frac{1}{n}L\left(\theta |y\right)=\frac{1}{n}\sum_{i=1}^{n}\log g(y_{i}|\theta )$$

tends to $E_{\tilde{y}}\left[\log g\left(\tilde{y}|\theta \right)\right]$ by the law of large numbers, which suggests how we can estimate the second term of KL(f||g).

The model selection based on the K-L divergence is straightforward when all the operating models are fixed probability distributions, i.e., g(y|θ)=g(y). The model with the largest empirical log-likelihood $\sum_{i}\log g\left(y_{i}\right)$ is favored, when the observed data y are used as the test sample. However, when the distribution family $g(\tilde{y}|\theta )$ contains some unknown parameters θ, the direct comparison becomes no longer feasible. A typical strategy is to conduct the model fitting first, and then compare the operating models specified at the fitted parameters. In this case, the same data are indeed used twice—in both model fitting (as the training sample) and evaluation (as the test sample). Therefore, the in-sample log-likelihood is not optimal for the predictive modeling. For a desirable out-of-sample predictive performance, a common idea is to identify a bias correction term to rectify the over-estimation bias of the in-sample estimator, which is also the focus of this work.

In the frequentist setting, the general model selection procedure chooses candidate models specified by some point estimate $\hat{\theta }$ based on a certain statistical principle such as maximum likelihood. A considerable amount of theoretical research has addressed this problem by correcting for the bias of $\frac{1}{n}\sum_{i}\log g\left(y_{i}|\hat{\theta }\right)$ in estimation of $E_{\tilde{y}}\left[\log g\left(\tilde{y}|\hat{\theta }\right)\right]$ [1,9,10,11]. A nice review can be found in Burnham Anderson [12].

Since the introduction of the AIC [1], researchers have commonly applied frequentist model selection methods into Bayesian modeling. However, the differences in the underlying philosophies between Bayesian and frequentist statistical inference caution against such direct applications. There also have been a few attempts to specialize the K-L divergence for Bayesian model selection (see, for example, [5,13,14]) in the last century. These methods are limited either in the scope of methodology or computational feasibility, especially when the parameters of the Bayesian models are in high-dimensional hierarchical structures.

The seminal work of Spiegelhalter et al. [6,15] proposed DIC,

$$DIC=D\left(\bar{\theta }\right)+2p_{D}$$

as a Bayesian adaption of AIC and implemented it using Gibbs sampling (BUGS) [16], where D(θ) is the deviance function, $\bar{\theta }$ is the posterior mean and $p_{D}$ is the effective number of parameters. Although its establishment lacks a theoretical foundation [17,18], −dic/2n, as a model selection measure, heuristically estimates $E_{\tilde{y}}\left[\log g\left(\tilde{y}|\bar{\theta }\right)\right]$, the expected out-of-sample log-likelihood specified at the posterior mean, after assuming that the proposed model encompasses the true model. Alternative methods can be found either using a similar approach for mixed-effects models [19,20,21] or using numerical approximation [22] to estimate cross-validative predictive loss [23].

## 3. Posterior Averaging Information Criterion

The preceding methods in general can be viewed as Bayesian adaptation of the information criteria originally designed for frequentist statistics, when each model is assessed in terms of the similarity between the true distribution *f* and the model density function specified by the plug-in parameters. This may not be ideal since, in contrast to frequentist modeling, “Bayesian inference is the process of fitting a probability model to a set of data and summarizing the result by *a probability distribution on the parameters* of the model and on unobserved quantities such as predictions for new observations” [8]. Rather than considering a model specified by a point estimate, it is more reasonable to assess the goodness of a Bayesian model in terms of the posterior distribution.

### 3.1. Rationale and the Proposed Method

Ando [7] proposed an estimator for the posterior averaged discrepancy function,

$$\eta =E_{\tilde{y}}\left[E_{\theta |y}\log g\left(\tilde{y}|\theta \right)\right].$$

Under certain regularity conditions, it was shown that an asymptotic unbiased estimator of η is

(2) $$\begin{array}{ccc} {\hat{\eta }}^{BPIC} & = & \frac{1}{n}E_{\theta |y}\log L\left(\theta |y\right)-\frac{1}{n}[\;E_{\theta |y}\log\left\{\pi \left(\theta \right)L\left(\theta |y\right)\right\}-\log\left\{\pi \left(\hat{\theta }\right)L\left(\hat{\theta }|y\right)\right\}\\ & & +tr\left\{J_{n}^{-1}\left(\hat{\theta }\right)I_{n}\left(\hat{\theta }\right)\right\}+\frac{K}{2}]\\ & ≜ & \frac{1}{n}E_{\theta |y}\log L\left(\theta |y\right)-BC_{1}. \end{array}$$

Here, π(θ) is the prior distribution, $\hat{\theta }$ is the posterior mode, *K* is the cardinality of θ, and matrices $J_{n}$ and $I_{n}$ are some empirical estimators for the Bayesian asymptotic Hessian matrix,

$$J\left(\theta \right)=-E_{\tilde{y}}\left(\frac{\partial^{2}\log\left\{g\left(\tilde{y}|\theta \right)\pi_{0}\left(\theta \right)\right\}}{\partial \theta \partial \theta^{\prime }}\right)$$

and Bayesian asymptotic Fisher information matrix,

$$I\left(\theta \right)=E_{\tilde{y}}\left(\frac{\partial \log\{g\left(\tilde{y}|\theta \right)\pi_{0}\left(\theta \right)\}}{\partial \theta }\frac{\partial \log\{g\left(\tilde{y}|\theta \right)\pi_{0}\left(\theta \right)\}}{\partial \theta^{\prime }}\right),$$

where $\log\pi_{0}\left(\theta \right)=\lim_{n\to \infty }n^{-1}\log\pi \left(\theta \right)$.

The Bayesian predictive information criterion (BPIC) was introduced as $-2n\cdot {\hat{\eta }}^{BPIC}$. It is applicable when the true model *f* is not necessarily in the specified family of probability distributions. In model comparison, the candidate model with a minimum BPIC value is favored. However, it has the following limitations in practice.

1. Equation (2) was from the original presentation for BPIC in Equation (5) of Ando [7]. After some math canceling out the term $\frac{1}{n}E_{\theta |y}\log L\left(\theta |y\right)$ in both estimator and bias correction term, ${\hat{\eta }}^{BPIC}$ can be simplified as

(3) $$\begin{array}{ccc} {\hat{\eta }}^{BPIC} & = & \frac{1}{n}\log L\left(\hat{\theta }|y\right)-\frac{1}{n}\left[\;E_{\theta |y}\log\pi \left(\theta \right)-\log\pi \left(\hat{\theta }\right)+tr\left\{J_{n}^{-1}\left(\hat{\theta }\right)I_{n}\left(\hat{\theta }\right)\right\}+\frac{K}{2}\right]\\ & ≜ & \frac{1}{n}\log L\left(\hat{\theta }|y\right)-BC_{2}, \end{array}$$

which shows that it was actually the plug-in estimator $\frac{1}{n}\log L\left(\hat{\theta }|y\right)$, rather than natural estimator $\frac{1}{n}E_{\theta |y}\log L\left(\theta |y\right)$, was used in estimation of η for bias correction. Compared with the natural estimator, the estimation efficiency of η using plug-in estimator is suboptimal when the posterior distribution is asymmetric.

2. The BPIC cannot be calculated when the prior distribution π(θ) is degenerate, a common situation in Bayesian analysis when an objective non-informative prior is selected. For example, if we use non-informative prior π(μ)∝1 for the mean parameter μ of the normal distribution in the following Section 4.1, the values of $\log\pi (\hat{\theta })$ and $E_{\theta |y}\log\pi \left(\theta \right)$ in Equation (3) are undefined.

In order to avoid those drawbacks, we propose a new model selection criterion in terms of the posterior mean of the empirical log-likelihood $\hat{\eta }=\frac{1}{n}E_{\theta |y}\log L\left(\theta |y\right)=\frac{1}{n}\sum_{i}E_{\theta |y}\left[\log g\left(y_{i}|\theta \right)\right]$, a natural estimator of estimand η. Without losing any of the attractive properties of BPIC, the new criterion expands the model scope to all regular Bayesian models. As we will show in the simulation study, empirically it also improves the unbiased property for small samples, and enhances the robustness of the estimation.

Because all the data y are used for both model fitting and model selection, $\hat{\eta }$ always overestimates η. To correct the estimation bias from the overuse of the data, we have the following theorem.

**Theorem 1.**  *Let $y=(y_{1},y_{2},\cdots ,y_{n})$ be n independent observations drawn from the probability cumulative distribution $F(\tilde{y})$ with density function $f(\tilde{y})$. Consider $G=\{g\left(\tilde{y}|\theta \right);\theta \in \Theta \subseteq R^{p}\}$ as a family of candidate statistical models that do not necessarily contain the true distribution f, where $\theta ={\left(\theta_{1},\ldots ,\theta_{p}\right)}^{\prime }$ is the p-dimensional vector of unknown parameters, with prior distribution π(θ). Under the following three regularity conditions:*

*C1:*  *Both the log density function $\log g(\tilde{y}|\theta )$ and the log unnormalized posterior density log{L(θ|y)π(θ)} are twice continuously differentiable in the compact parameter space *Θ*;*
*C2:*  *The expected posterior mode $\theta_{0}=\arg\max_{\theta }E_{\tilde{y}}\left[\log\left\{g\left(\tilde{y}|\theta \right)\pi_{0}\left(\theta \right)\right\}\right]$ is unique in *Θ*;*
*C3:*  *The Hessian matrix of $E_{\tilde{y}}\left[\log\left\{g\left(\tilde{y}|\theta \right)\pi_{0}\left(\theta \right)\right\}\right]$ is non-singular at $\theta_{0}$,*

*the bias of $\hat{\eta }$ for η can be approximated asymptotically without bias by*

(4) $$\hat{\eta }-\eta =\hat{b_{\theta }}≅\frac{1}{n}tr\left\{J_{n}^{-1}\left(\hat{\theta }\right)I_{n}\left(\hat{\theta }\right)\right\},$$

*where $\hat{\theta }$ is the posterior mode that maximizes the posterior distribution $\propto \pi \left(\theta \right)\prod_{i=1}^{n}g\left(y_{i}|\theta \right)$ and*

$$\begin{array}{ccc} J_{n}\left(\theta \right) & = & -\frac{1}{n}\sum_{i=1}^{n}\left(\frac{\partial^{2}\log\left\{g\left(y_{i}|\theta \right)\pi^{\frac{1}{n}}\left(\theta \right)\right\}}{\partial \theta \partial \theta^{\prime }}\right)\\ I_{n}\left(\theta \right) & = & \frac{1}{n-1}\sum_{i=1}^{n}\left(\frac{\partial \log\{g\left(y_{i}|\theta \right)\pi^{\frac{1}{n}}\left(\theta \right)\}}{\partial \theta }\frac{\partial \log\{g\left(y_{i}|\theta \right)\pi^{\frac{1}{n}}\left(\theta \right)\}}{\partial \theta^{\prime }}\right). \end{array}$$

**Proof.**  Recall that the quantity of interest is $E_{\tilde{y}}E_{\theta |y}\log g\left(\tilde{y}|\theta \right)$. To estimate it, we first check

$E_{\tilde{y}}E_{\theta |y}\log\left\{g\left(\tilde{y}|\theta \right)\pi_{0}\left(\theta \right)\right\}=E_{\tilde{y}}E_{\theta |y}\left\{\log g\left(\tilde{y}|\theta \right)+\log\pi_{0}\left(\theta \right)\right\}$ and expand it about $\theta_{0}$,

(5) $$\begin{array}{ccc} E_{\tilde{y}}E_{\theta |y}\log\left\{g\left(\tilde{y}|\theta \right)\pi_{0}\left(\theta \right)\right\} & = & E_{\tilde{y}}\log\left\{g\left(\tilde{y}|\theta_{0}\right)\pi_{0}\left(\theta_{0}\right)\right\}+E_{\theta |y}{\left(\theta -\theta_{0}\right)}^{\prime }\frac{\partial E_{\tilde{y}}\log\left\{g\left(\tilde{y}|\theta \right)\pi_{0}\left(\theta \right)\right\}}{\partial \theta }{|}_{\theta =\theta_{0}}\\ & & +\frac{1}{2}E_{\theta |y}\left[{\left(\theta -\theta_{0}\right)}^{\prime }\frac{\partial^{2}E_{\tilde{y}}\log\left\{g\left(\tilde{y}|\theta \right)\pi_{0}\left(\theta \right)\right\}}{\partial \theta \partial \theta^{\prime }}{|}_{\theta =\theta_{0}}\left(\theta -\theta_{0}\right)\right]+o_{p}\left(n^{-1}\right)\\ & = & E_{\tilde{y}}\log\left\{g\left(\tilde{y}|\theta_{0}\right)\pi_{0}\left(\theta_{0}\right)\right\}+E_{\theta |y}{\left(\theta -\theta_{0}\right)}^{\prime }\frac{\partial E_{\tilde{y}}\log\left\{g\left(\tilde{y}|\theta \right)\pi_{0}\left(\theta \right)\right\}}{\partial \theta }{|}_{\theta =\theta_{0}}\\ & & -\frac{1}{2}E_{\theta |y}\left[{\left(\theta -\theta_{0}\right)}^{\prime }J\left(\theta_{0}\right)\left(\theta -\theta_{0}\right)\right]+o_{p}\left(n^{-1}\right)\\ & ≜ & I_{1}+I_{2}+I_{3}+o_{p}\left(n^{-1}\right) \end{array}$$

The first term $I_{1}$ can be linked to the empirical log likelihood function as follows:

$$\begin{array}{ccc} E_{\tilde{y}}\log\left\{g\left(\tilde{y}|\theta_{0}\right)\pi_{0}\left(\theta_{0}\right)\right\} & = & E_{\tilde{y}}\log g\left(\tilde{y}|\theta_{0}\right)+\log\pi_{0}\left(\theta_{0}\right)\\ & = & E_{y}\frac{1}{n}\log L\left(\theta_{0}|y\right)+\log\pi_{0}\left(\theta_{0}\right)\\ & = & E_{y}\frac{1}{n}\log\left\{L\left(\theta_{0}|y\right)\pi \left(\theta_{0}\right)\right\}-\frac{1}{n}\log\pi \left(\theta_{0}\right)+\log\pi_{0}\left(\theta_{0}\right)\\ & = & E_{y}E_{\theta |y}\frac{1}{n}\log\left\{L\left(\theta |y\right)\pi \left(\theta \right)\right\}-\frac{1}{2n}tr\left\{J_{n}^{-1}\left(\theta_{0}\right)I\left(\theta_{0}\right)\right\}\\ & & +\frac{1}{2n}tr\left\{J_{n}^{-1}\left(\hat{\theta }\right)J_{n}\left(\theta_{0}\right)\right\}-\frac{1}{n}\log\pi \left(\theta_{0}\right)+\log\pi_{0}\left(\theta_{0}\right)+o_{p}\left(n^{-1}\right) \end{array}$$

where the last equation holds due to Lemma A5 (together with other Lemmas, provided in the Appendix A).

The second term $I_{2}$ vanishes since

$$\frac{\partial E_{\tilde{y}}\log\left\{g\left(\tilde{y}|\theta \right)\pi_{0}\left(\theta \right)\right\}}{\partial \theta }{|}_{\theta =\theta_{0}}=0$$

as $\theta_{0}$ is the expected posterior mode.

Using Lemma A4, the third term $I_{3}$ can be rewritten as

$$\begin{array}{ccc} I_{3} & = & -\frac{1}{2}E_{\theta |y}{\left(\theta -\theta_{0}\right)}^{\prime }J\left(\theta_{0}\right)\left(\theta -\theta_{0}\right)\\ & = & -\frac{1}{2}tr\left\{E_{\theta |y}\left[\left(\theta -\theta_{0}\right){\left(\theta -\theta_{0}\right)}^{\prime }\right]J\left(\theta_{0}\right)\right\}\\ & = & -\frac{1}{2n}\left(tr\left\{J_{n}^{-1}\left(\theta_{0}\right)I\left(\theta_{0}\right)J_{n}^{-1}\left(\theta_{0}\right)J\left(\theta_{0}\right)\right\}+tr\left\{J_{n}^{-1}\left(\hat{\theta }\right)J\left(\theta_{0}\right)\right\}\right)+o_{p}\left(n^{-1}\right) \end{array}$$

By substituting each term in Equation (5) and neglecting the residual term, we obtain

$$\begin{array}{ccc} E_{\tilde{y}}E_{\theta |y}\log\left\{g\left(\tilde{y}|\theta \right)\pi_{0}\left(\theta \right)\right\} & ≃ & E_{y}E_{\theta |y}\frac{1}{n}\log\left\{L\left(\theta |y\right)\pi \left(\theta \right)\right\}-\frac{1}{2n}tr\left\{J_{n}^{-1}\left(\theta_{0}\right)I\left(\theta_{0}\right)\right\}\\ & & +\frac{1}{2n}tr\left\{J_{n}^{-1}\left(\hat{\theta }\right)J_{n}\left(\theta_{0}\right)\right\}-\frac{1}{n}\log\pi \left(\theta_{0}\right)+\log\pi_{0}\left(\theta_{0}\right)\\ & & -\frac{1}{2n}\left(tr\left\{J_{n}^{-1}\left(\theta_{0}\right)I\left(\theta_{0}\right)J_{n}^{-1}\left(\theta_{0}\right)J\left(\theta_{0}\right)\right\}+tr\left\{J_{n}^{-1}\left(\hat{\theta }\right)J\left(\theta_{0}\right)\right\}\right) \end{array}$$

Recall that we have defined $\log\pi_{0}\left(\theta \right)=\lim_{n\to \infty }n^{-1}\log\pi \left(\theta \right)$, so that asymptotically we have

$$\begin{array}{cc} & \log\pi_{0}\left(\theta_{0}\right)-\frac{1}{n}\log\pi \left(\theta_{0}\right)≃0,\\ & E_{\theta |y}\log\left\{\pi_{0}\left(\theta \right)\right\}-E_{\theta |y}\frac{1}{n}\log\left\{\pi \left(\theta \right)\right\}≃0. \end{array}$$

Therefore, $E_{\tilde{y}}E_{\theta |y}\log\left\{g\left(\tilde{y}|\theta \right)\right\}$ can be estimated by

$$\begin{array}{ccc} E_{\tilde{y}}E_{\theta |y}\log\left\{g\left(\tilde{y}|\theta \right)\right\} & = & E_{\tilde{y}}E_{\theta |y}\log\left\{g\left(\tilde{y}|\theta \right)\pi_{0}\left(\theta \right)\right\}-E_{\theta |y}\log\left\{\pi_{0}\left(\theta \right)\right\}\\ & ≃ & E_{y}E_{\theta |y}\frac{1}{n}\log\left\{L\left(\theta |y\right)\pi \left(\theta \right)\right\}-\frac{1}{2n}tr\left\{J_{n}^{-1}\left(\theta_{0}\right)I\left(\theta_{0}\right)\right\}+\frac{1}{2n}tr\left\{J_{n}^{-1}\left(\hat{\theta }\right)J_{n}\left(\theta_{0}\right)\right\}\\ & & -\frac{1}{2n}\left(tr\left\{J_{n}^{-1}\left(\theta_{0}\right)I\left(\theta_{0}\right)J_{n}^{-1}\left(\theta_{0}\right)J\left(\theta_{0}\right)\right\}+tr\left\{J_{n}^{-1}\left(\hat{\theta }\right)J\left(\theta_{0}\right)\right\}\right)\\ & & -\frac{1}{n}\log\pi \left(\theta_{0}\right)+\log\pi_{0}\left(\theta_{0}\right)-E_{\theta |y}\log\left\{\pi_{0}\left(\theta \right)\right\}\\ & ≃ & E_{y}E_{\theta |y}\frac{1}{n}\log\left\{L\left(\theta |y\right)\right\}-\frac{1}{2n}tr\left\{J_{n}^{-1}\left(\theta_{0}\right)I\left(\theta_{0}\right)\right\}+\frac{1}{2n}tr\left\{J_{n}^{-1}\left(\hat{\theta }\right)J_{n}\left(\theta_{0}\right)\right\}\\ & & -\frac{1}{2n}\left(tr\left\{J_{n}^{-1}\left(\theta_{0}\right)I\left(\theta_{0}\right)J_{n}^{-1}\left(\theta_{0}\right)J\left(\theta_{0}\right)\right\}+tr\left\{J_{n}^{-1}\left(\hat{\theta }\right)J\left(\theta_{0}\right)\right\}\right) \end{array}$$

Replacing $\theta_{0}$ by $\hat{\theta }$, $J(\theta_{0})$ by $J_{n}\left(\hat{\theta }\right)$ and $I(\theta_{0})$ by $I_{n}\left(\hat{\theta }\right)$, we obtain $E_{\theta |y}\frac{1}{n}\log\left\{L\left(\theta |y\right)\right\}-\frac{1}{n}tr\left\{J_{n}^{-1}\left(\hat{\theta }\right)I_{n}\left(\hat{\theta }\right)\right\}$ as an asymptotically unbiased estimate for $E_{\tilde{y}}E_{\theta |y}\log\left\{g\left(\tilde{y}|\theta \right)\right\}$. □

With the above result, we propose a new predictive criterion for Bayesian modeling, named the Posterior Averaging Information Criterion (PAIC),

(6) $$PAIC=-2\sum_{i}E_{\theta |y}\left[\log g\left(y_{i}|\theta \right)\right]+2tr\left\{J_{n}^{-1}\left(\hat{\theta }\right)I_{n}\left(\hat{\theta }\right)\right\}.$$

The candidate models with small criterion values (6) are preferred for the purpose of model selection.

**Remark 1.**  *PAIC selects the candidate models with optimal performance to predict a future outcome.*

The optimality is defined in a sense to maximize the out-of-sample log density η, which is equivalent to minimize the posterior predictive K-L divergence.

**Remark 2.**  *PAIC is derived without assuming that the approximating distributions contain the truth.*

In another word, PAIC is generally applicable even if all candidate models are misspecified. In such settings, rather than select the true model, the goal is to identify the best candidate model(s) with small PAICs among all models under consideration. Similar to other K-L based information criteria, we consider a model is better if its PAIC is smaller with a difference larger than 2.

**Remark 3.**  *The averaging over the posterior distribution in empirical likelihood helps differentiate the candidate models.*

The posterior distribution function, rather than a point estimator, represents the current best knowledge from a Bayesian perspective. In some cases, two candidate models may have identical posterior mean but different posterior distributions. (A simple example could be in the setting of Section 4.1, when model A has $\tau_{0}=1000$ and model B has $\tau_{0}=1$ in the prior distribution.) Apparently, Bayesian model assessment with respect to the posterior distribution is more effective in model selection. When the posterior distribution of the parameters is asymmetric, the estimation of information criterion averaged over the posterior is also more robust than plugging in a point estimator.

**Remark 4.**  *PAIC can be applied to Bayesian models with flexible prior structures.*

For example, in cases when the prior distributions are consistent and sample size dependent [24,25], the information in the prior distribution does not degenerate asymptotically, but is accommodated spontaneously in empirical log-likelihood and bias-correction for predictive assessment. Unlike the BPIC, PAIC relaxes the restriction in common prior distribution specification. It is well-defined and can cope with degenerate non-informative prior distributions for parameters. The bias correction term $tr\{J_{n}^{-1}\left(\hat{\theta }\right)I_{n}\left(\hat{\theta }\right)\}$ is closely related to the concept of measuring a Bayesian model’s complexity [26]. Particularly, when the candidate model is true and has no hierarchical structures, and the prior distribution is non-informative with a dimension of *p*, we have exactly $tr\{J_{n}^{-1}\left(\hat{\theta }\right)I_{n}\left(\hat{\theta }\right)\}=p$, which is similar to the bias correction in AIC [1].

### 3.2. Relevant Methods for the Posterior Averaged K-L Discrepancy

Rather than deriving the bias correction analytically, resampling approaches, such as cross-validation and bootstrap, can also be used to measure the posterior averaged K-L discrepancy. Plummer [22] introduced the expected deviance penalized loss with ‘expected deviance’ defined as

$$L^{e}\left(y_{i},z\right)=-2E_{\theta |z}\log g(y_{i}|\theta ),$$

which is a special case of the predictive discrepancy measure [27]. The standard cross-validation method can also be applied in this circumstance to estimate η, simply by considering the K-L discrepancy as the utility function of [28] and further investigated by [29]. The estimation of the bootstrap error correction $\eta^{\left(b\right)}-{\hat{\eta }}^{\left(b\right)}$ with bootstrap analogues

$$\eta^{\left(b\right)}=E_{\tilde{y^{*}}}\left[E_{\theta |y^{*}}\log g\left(\tilde{y}|\theta \right)\right]$$

and

$${\hat{\eta }}^{\left(b\right)}=E_{\tilde{y^{*}}}\left[n^{-1}E_{\theta |y^{*}}\log L\left(\theta |y^{*}\right)\right]$$

for $\eta -\hat{\eta }$ was discussed by Ando [7] as a Bayesian adaptation of frequentist model selection [10]. Although numeric algorithms such as importance sampling can be used for intensive computation, one caveat is that it may cause inaccurate estimation in practice if some observation $y_{i}$ was influential [28]. To address that problem, Vehtari [30] proposed Pareto smoothed importance sampling, a new algorithm for regularizing importance weights, and developed a numerical tool [31] to facilitate computation. Watanabe [32] established a singular learning theory and proposed a new criterion named Watanabe–Akaike [29], or widely applicable information criterion (WAIC) [33,34], while WAIC${}_{1}$ was proposed for the plug-in discrepancy and WAIC${}_{2}$ for the posterior averaged discrepancy. However, compared with BPIC and PAIC, we found that WAIC${}_{2}$ tends to have a larger bias and variation for regular Bayesian models, as shown in simulation studies in the next section.

## 4. Simulation Study

In this section, we present some numerical results to illustrate the performance of the proposed method under small sample sizes. Assuming K-L divergence is a good measure for model selection, our goal is simply to assess how it can be estimated with the smallest bias. In the simulation experiments, we estimate the true expected bias η either analytically in a Gaussian setting (Section 4.1) or numerically by averaging $E_{\theta |y}\left[\log g\left(\tilde{y}|\theta \right)\right]$ over a large number of extra independent draws of $\tilde{y}$ when there is asymmetric posterior distribution and no closed form for the integration (Section 4.2). To have BPIC well-defined for comparison, only the proper prior distributions are considered.

### 4.1. A Case with Closed-Form Expression for Bias Estimators

Suppose observations $y=(y_{1},y_{2},\ldots ,y_{n})$ are a vector of iid samples generated from $N(\mu_{T},\sigma_{T}^{2})$, with unknown true mean $\mu_{T}$ and variance $\sigma_{T}^{2}=1$. Assume the data are analyzed by the approximating model $g\left(y_{i}|\mu \right)=N\left(\mu ,\sigma_{A}^{2}\right)$ with prior $\pi \left(\mu \right)=N\left(\mu_{0},\tau_{0}^{2}\right)$, where $\sigma_{A}^{2}$ is fixed, but not necessarily equal to the true variance $\sigma_{T}^{2}$. When $\sigma_{A}^{2}\neq \sigma_{T}^{2}$, the model is misspecified.

The posterior distribution of μ is normally distributed with mean $\hat{\mu }$ and variance ${\hat{\sigma }}^{2}$, where

$$\begin{array}{ccc} \hat{\mu } & = & \left(\mu_{0}/\tau_{0}^{2}+\sum_{i=1}^{n}y_{i}/\sigma_{A}^{2}\right)/\left(1/\tau_{0}^{2}+n/\sigma_{A}^{2}\right)\\ {\hat{\sigma }}^{2} & = & 1/(1/\tau_{0}^{2}+n/\sigma_{A}^{2}). \end{array}$$

Therefore, the K-L discrepancy function and its estimator are

$$\begin{array}{ccc} \eta & = & E_{\tilde{y}}\left[E_{\mu |y}\left[\log g\left(\tilde{y}|\mu \right)\right]\right]=-\frac{1}{2}\log\left(2\pi \sigma_{A}^{2}\right)-\frac{\sigma_{T}^{2}+{\left(\mu_{T}-\hat{\mu }\right)}^{2}+{\hat{\sigma }}^{2}}{2\sigma_{A}^{2}}\\ \hat{\eta } & = & \frac{1}{n}\sum_{i=1}^{n}E_{\mu |y}\left[\log g\left(y_{i}|\mu \right)\right]]=-\frac{1}{2}\log\left(2\pi \sigma_{A}^{2}\right)-\frac{1}{n}\sum_{i=1}^{n}\frac{{\left(y_{i}-\hat{\mu }\right)}^{2}+{\hat{\sigma }}^{2}}{2\sigma_{A}^{2}}. \end{array}$$

We assess the bias estimator defined in Theorem 1, ${\hat{b}}_{\mu }^{PAIC}$ and four other bias estimators: ${\hat{b}}_{\mu }^{BPIC}$[7], ${\hat{b}}_{\mu }^{WAIC_{2}}$[33], ${\hat{b}}_{\mu }^{p_{opt}}$[22], and ${\hat{b}}_{\mu }^{CV}$ [35].

$$\begin{array}{ccc} {\hat{b}}_{\mu }^{PAIC} & = & \frac{1}{n-1}{\hat{\sigma }}^{2}\sum_{i=1}^{n}{\left(\left(\mu_{0}-\hat{\mu }\right)/\left(n\tau_{0}^{2}\right)+\left(y_{i}-\hat{\mu }\right)/\sigma_{A}^{2}\right)}^{2}\\ {\hat{b}}_{\mu }^{BPIC} & = & \frac{1}{n}{\hat{\sigma }}^{2}\sum_{i=1}^{n}{\left(\left(\mu_{0}-\hat{\mu }\right)/\left(n\tau_{0}^{2}\right)+\left(y_{i}-\hat{\mu }\right)/\sigma_{A}^{2}\right)}^{2}\\ {\hat{b}}_{\mu }^{WAIC_{2}} & = & \frac{{\hat{\sigma }}^{2}}{\sigma_{A}^{4}}\left(n{\hat{\sigma }}^{2}/2+\sum_{i=1}^{n}{\left(y_{i}-\hat{\mu }\right)}^{2}\right)\\ {\hat{b}}_{\mu }^{p_{opt}} & = & \frac{1}{2n}p_{opt}=1/\left(1/\tau_{0}^{2}+\left(n-1\right)/\sigma_{A}^{2}\right)/\sigma_{A}^{2}\\ {\hat{b}}_{\mu }^{CV} & = & \hat{\eta }-\left(\sum_{i=1}^{n}{\left(y_{i}-\left(\mu_{0}/\tau_{0}^{2}+\sum_{j\neq i}y_{j}/\sigma_{A}^{2}\right)/\left(1/\tau_{0}^{2}+\left(n-1\right)/\sigma_{A}^{2}\right)\right)}^{2}/n+{\hat{\sigma }}^{2}\right)/\sigma_{A}^{2}/2. \end{array}$$

We compare them with the true bias

$$b_{\mu }=E_{y}\left(\hat{\eta }-\eta \right)=E_{y}\left\{\frac{\sigma_{T}^{2}}{2\sigma_{A}^{2}}+\frac{{\left(\mu_{T}-\hat{\mu }\right)}^{2}}{2\sigma_{A}^{2}}-\frac{1}{n}\sum_{i=1}^{n}\frac{{\left(y_{i}-\hat{\mu }\right)}^{2}}{2\sigma_{A}^{2}}\right\}=\sigma_{T}^{2}{\hat{\sigma }}^{2}/\sigma_{A}^{4}.$$

**Figure 1:**
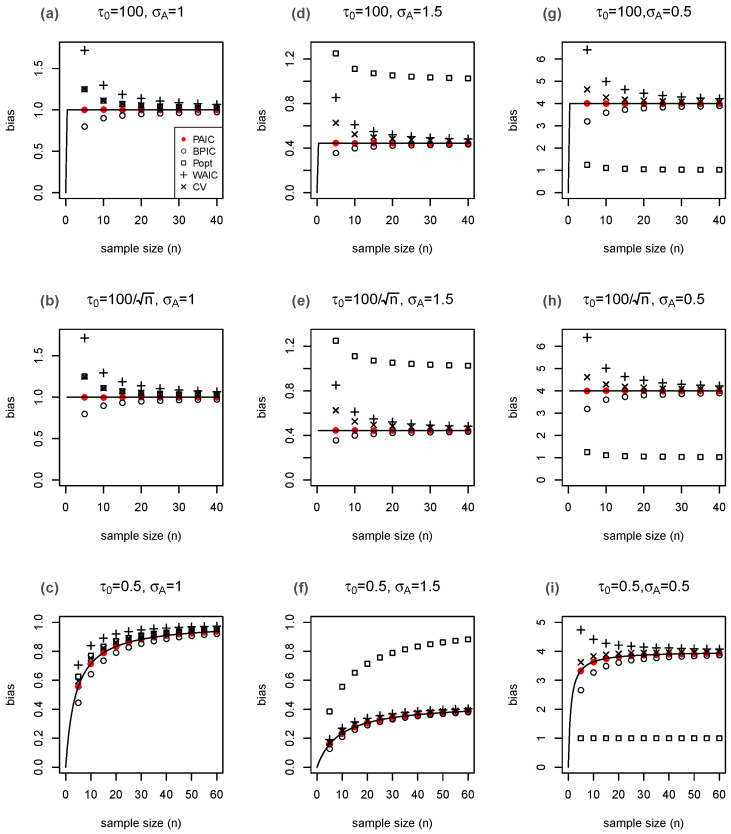
Performance of the bias estimators for $n\times E_{y}\left(\hat{\eta }-\eta \right)$. The top panels are under a relatively non-informative prior with $\tau_{0}^{2}=10^{4}$; the middle panels are under the case that the prior distribution grows with sample size with $\tau_{0}^{2}=10^{4}/n$; the bottom panels are under an informative prior with $\tau_{0}^{2}=0.25$. The left panels (**a**–**c**) are under the scenario of $\sigma_{A}^{2}=\sigma_{T}^{2}=1$, i.e., the true distribution is contained in the candidate models. The middle panels (**d**–**f**) are under the scenario of $\sigma_{A}^{2}=2.25$ and right panels (**g**–**i**) are under the scenario of $\sigma_{A}^{2}=0.25$ when the proposed model is misspecified from $\sigma_{T}^{2}=1$. The true bias $b_{\mu }$ is curved by (—) as a function of sample size *n*. The averages of the different bias estimators are marked by (•) for PAIC; (∘) for BPIC; (□) for $p_{opt}$; (+) for WAIC${}_{2}$; and (×) for cross-validation. Each mark represents the mean of the estimated bias of 100,000 replications of y.

The results are in accordance with the theory (Figure 1). All of the estimates are close to the true bias-correction values when the model is well-specified with $\sigma_{A}^{2}=\sigma_{T}^{2}=1$, especially when the sample size becomes moderately large (Figure 1, panels (a), (b), and (c)). The estimated values based on the PAIC are consistently closer to the true values than either those based on Ando’s method, which underestimates the bias, or the WAIC${}_{2}$, cross-validation or expected deviance penalized loss, which overestimate the bias, especially when the sample size is small. When the models are misspecified, it is not surprising that in all of the plots given in panels (d)–(i) of Figure 1, only the expected deviance penalized loss misses the target even asymptotically since its assumption is violated, whereas all the other approaches converge to $b_{\mu }$. In summary, PAIC achieves the best overall performance.

### 4.2. Bayesian Logistic Regression

Consider frequencies $y=\{y_{1},\ldots ,y_{N}\}$, which are independent observations from binomial distributions with respective true probabilities $\xi_{1}^{T},\ldots ,\xi_{N}^{T}$, and sample sizes, $n_{1},\ldots ,n_{N}$. To draw the inference of the ξ’s, we assume that the logits

$$\beta_{i}=\mathrm{logit}\left(\xi_{i}\right)=\log\frac{\xi_{i}}{1-\xi_{i}}$$

are random effects that follow the normal distribution $\beta_{i}∼N\left(\mu ,\tau^{2}\right).$ The weakly-informative joint prior distribution $N(\mu ;0,1000^{2})\cdot Inv$-$\chi^{2}\left(\tau^{2};0.1,10\right)$ is proposed on the hyper-parameter $\left(\mu ,\tau^{2}\right)$ so that the BPIC is properly defined and computable. The posterior distribution is asymmetric due to the logistic transformation.

We compare the performance of four asymptotically unbiased bias estimators in this hierarchical, asymmetric setting. The true bias η does not have an analytical form. We estimate it through numerical computation using independent simulation from the same data generating process, assuming the underlying true values of μ=0 and τ=1. The simulation scheme is as follows:

1. Draw $\beta_{T,i}∼N\left(0,1\right)$, $y_{i}∼Bin\left(n_{i},{\mathrm{logit}}^{-1}\left(\beta_{T,i}\right)\right)$, i=1,…,N from the true distribution.
2. Simulate the posterior draws of (β,μ,τ)|y.
3. Estimate ${\hat{b}}_{\beta }^{PAIC}$, ${\hat{b}}_{\beta }^{BPIC}$, ${\hat{b}}_{\beta }^{WAIC_{2}}$, and ${\hat{b}}_{\beta }^{CV}$.
4. Draw $z^{\left(j\right)}∼Bin\left(n,{\mathrm{logit}}^{-1}\left(\beta_{0}^{T}\right)\right)$, j=1,…,J, for approximation of true η.
5. Compare each ${\hat{b}}_{\beta }$ with true bias $b_{\beta }=\hat{\eta }-\eta$.
6. Repeat steps 1–5.

Table 1 summarizes the bias and standard deviation of the estimation error when we choose N=15 and $n_{1}=\ldots =n_{N}=50$, and the β’s are independently simulated from the standard normal distribution assuming the true hyper-parameter mean μ=0 and variance $\tau^{2}=1$. The simulation is repeated for 1000 scenarios, each with J= 20,000 for out-of-sample η estimation. PAIC and BPIC were calculated based on definition; leave-one-out cross-validation and WAIC${}_{2}$ were estimated using *R* package *loo* v2.5.1 [31]. The actual error, mean absolute error, and mean square error were considered to assess the estimation error using the bias correction estimates. With respect to all three different metrics, the bias estimation of PAIC is consistently superior to other methods. Compared to BPIC, the second best performed model selection criterion, the bias, and the mean squared error of PAIC are reduced by about 40%, while the absolute bias is reduced by about one quarter, which matches our expectation that the natural estimate $\frac{1}{n}\sum_{i}E_{\theta |y}\left[\log g\left(y_{i}|\theta \right)\right]$ will estimate the posterior averaged K-L discrepancy more precisely than plug-in estimate $\frac{1}{n}\sum_{i}\log g\left(y_{i}|\hat{\theta }\right)$ when the posterior distribution is asymmetric and correlated. Compared to WAIC${}_{2}$, the bias, absolute error, and mean square error of PAIC are dramatically reduced by at least 60%. In practice, we expect the improvement is even larger when proposed models have more complicated hierarchical structures.

As suggested by reviewers, we also assessed PAIC in bias estimation with different priors, including the commonly used Inv-$Gamma^{2}\left(\tau^{2};0.001,0.001\right)$[36]. Although these priors may produce different posterior distributions, we found almost identical results in terms of bias estimation error to Table 1, suggesting the robustness of the proposed method. Furthermore, we examined the BPIC and PAIC for uncorrelated posterior distributions of βs, by fixing the hyperparameters $\left(\mu ,\tau^{2}\right)$ either at its true value or at the posterior mode. In the simulation replications containing extreme observations (i.e., ∃i∈{1,…,N}, such that either $y_{i}=0$ or $y_{i}=n_{i}$), we observed a large deviation of the plug-in estimate $1/N\log L(\hat{\theta }|y)$ to η, which cannot be properly recovered by BPIC’s bias correction term in Equation (3) and yields significant estimation error; meanwhile, the plug-in estimand $E_{\tilde{y}}\left[\log g\left(\tilde{y}|\hat{\beta }\right)\right]$ was also much more vulnerable to the observed data than $\eta =E_{\tilde{y}}\left[E_{\beta |y}\log g\left(\tilde{y}|\beta \right)\right]$ given the extreme value, suggesting that the latter (the posterior averaged discrepancy) could be a better choice for model assessment.

## 5. Application

This is a variable selection example that uses real data to illustrate the practical difference between criteria proposed in either the explanatory or predictive perspective. We explore the problem of finding the best model to predict the selling of new accounts in branches of a large bank. The data were introduced in example 5.3 of George & McCulloch [37], analyzed with their method, the stochastic search variable selection (SSVS) technique to select the promising subsets of predictors. Their report on the 10 most frequently selected models after 10,000 iterations of Gibbs sampling for potential subsets, is listed in the first column of Table 2.

The original data consist of the numbers of new accounts sold in some time period as the outcome y, together with 15 predictor variables *X* in each of 233 branches. Multiple linear regressions are employed to fit the data in the form of

$$y_{i}|\beta^{\left(m\right)},\sigma_{y}^{2}∼N\left(X^{\left(m\right)}\beta^{\left(m\right)},\sigma_{y}^{2}\right)$$

with prior $\beta_{i}^{\left(m\right)}∼N\left(0,1000^{2}\right)$ and $\sigma_{y}^{2}∼Inv-Gamma\left(0.001,0.001\right)$, when *m* indicates the specific model with a subset of predictor $X^{\left(m\right)}$.

Several model selection estimators for −2n·η, including the leave-one-out cross-validated estimator (LOO-CV), *K*-fold cross-validated estimator (KCV), the expected deviance penalized loss with $p_{opt}^{e}$, BPIC, and PAIC, are calculated based on a large number of MCMC draws of the posterior distribution for model selection inference. In KCV, the original data are randomly partitioned for the *K*-fold cross-validation with a common choice of K=10. All the posterior samples are simulated from three parallel chains based on MCMC techniques for model selection inference. To generate 15,000 effective draws of the posterior distribution, only one out of five iterations after convergence are kept to reduce the serial correlation.

The results are presented in Table 2, in which the models that have the smallest estimation value by each criterion are highlighted. The first 10 models with SSVS frequencies were originally picked by SSVS as shown in George & McCulloch [37]. An interesting finding is that the favored models selected by the K-L based criteria and SSVS are quite different. All of the K-L based criteria are developed in a predictive perspective, whereas SSVS is a variable selection method to pursue the model that best describes the given set of data. This illustrates that with different modeling purposes, either explanatory or predictive, the ‘best’ models found may not coincide. The estimated $PL_{p_{opt}^{e}}$, BPIC, and PAIC values for every candidate model are quite close to each other; whereas the cross-validation estimators are noisy due to the simulation error and tendency to overestimate the value. It is worth mentioning that the estimators of LOO-CV, K-fold-CV, and $PL_{p_{opt}^{e}}$ are relatively unstable, even with 15,000 posterior draws. Those methods have been much more computationally intensive than BPIC and PAIC.

## 6. Discussion

A clearly defined model selection criterion or score usually lies at the heart of any statistical selection and decision procedure. It facilitates the comparison of competing models through the assignment of some sort of preference or ranking to the alternatives. One of the typical scores is the K-L divergence, a non-symmetric measure of the difference between two probability distributions. By further acknowledging uncertainty in parameters and randomness in data, frequentist statistics theoretically employing K-L divergence into parametric model selection emerged during the 1970s. Since then, the development of related theories and applications has rapidly accelerated.

A good assessment measure helps establish attractive properties. To guide the Bayesian method development, two important questions should be first investigated.

1. What is a good estimand, based on K-L discrepancy, to evaluate Bayesian models?
2. What is a good estimator to estimate the estimand for K-L based Bayesian model selection?

The prevailing plug-in parameter methods, such as DIC, presume the candidate models are correct, and assess the goodness of each candidate model with a density function specified by the plug-in parameters. However, from a Bayesian perspective, it is inherent to examine the performance of a Bayesian model over the entire posterior distribution, as stated by (Celeux et al. [18], p. 703): “...we concede that using a plug-in estimate disqualifies the technique from being properly Bayesian.” Accordingly, statistical approaches to estimate the K-L discrepancy as evaluated by averaging over the posterior distribution are of great interest.

We have proposed PAIC, a versatile model selection technique for Bayesian models under regularity assumptions, to address this problem. From a predictive perspective, we consider the asymptotic unbiased estimation of a K-L discrepancy, which averages the conditional density of the observable data against the posterior knowledge about the unobservable data. Empirically, the proposed PAIC measures the similarity of the fitted model and the underlying true distribution, regardless of whether or not the approximating distribution family contains the true model. The range of applications of the proposed criterion can be quite broad.

PAIC and BPIC are similar in many aspects. In addition to all the asymptotic properties and similar computational costs both methods share, PAIC has some unique features, mainly because it employs the natural posterior-averaged estimator. For example, PAIC can be well applied even if the prior distribution of the parameters degenerates, in which case BPIC becomes uninterpretable. In the illustrative experiments, we focused on the comparison of estimation accuracy between the proposed criterion and other Bayesian model selection criteria including BPIC and WAIC${}_{2}$. PAIC showed the least bias and variance to estimate the posterior averaged discrepancy.

Because the regularity condition assumes twice continuously differentiability and non-singularity, it could be problematic if the posterior mode is on the boundary of the parameter space Θ. For example, as pointed out by one reviewer, $\hat{\tau }=0$ in the famous eight-school example [8]. This is a common concern for K-L based model selection since the method derivation relies on the Taylor series expansion. However, in practice, a reparameterization may help. In the eight-school example, we can introduce the uniform prior ϕ=logτ∼Unif(0,1) to pair with the weakly informative prior μ∼N(0,100), which yields a posterior mode for $\hat{\tau }=1.125$.

There are some future directions for the current work. In the current simulation setting, we made a default assumption that the estimand, i.e., the posterior-averaged out-of-sample log-likelihood, can be distinguished between candidate models. A more comprehensive comparison of Bayesian predictive methods for empirical model selection can be investigated by taking into account the likely over-fitting in the selection phase, similar to [38]. Because the users of PAIC and BPIC have to specify the first and second derivatives of the posterior distribution in their modeling, development of advanced computational tools for simultaneous calculations will be helpful. In singular learning machines, the regularity conditions can be relaxed to singular in a sense that the mapping from parameters to probability distributions is not necessarily one-to-one. Although here we focused on only the regular models, it is also possible to generalize PAIC to singular settings with a modified bias correction term, after an algebraic geometrical transformation of the singular parameter space to a real *d*-dimensional manifold. Finally, other metrics for comparing the distance or dissimilarity between two distributions, such as Hellinger distance [39] or Jensen–Shannon divergence [40], may be explored further and employed as alternative metrics in Bayesian model assessment.

## Figures and Tables

**Table 1:** The estimation error of bias correction: the mean and standard deviation (in parentheses) from 1000 replications.

Criterion	Actual Error	Mean Absolute Error	Mean Square Error
	$\hat{\eta }-\eta -{\hat{b}}_{\beta }$	$\left|\hat{\eta }-\eta -{\hat{b}}_{\beta }\right|$	${\left(\hat{\eta }-\eta -{\hat{b}}_{\beta }\right)}^{2}$
PAIC	**0.160 (0.238)**	**0.206 (0.199)**	**0.082 (0.207)**
BPIC	0.259 (0.244)	0.272 (0.229)	0.127 (0.267)
CV	0.840 (0.285)	0.840 (0.285)	0.786 (0.633)
$WAIC_{2}$	0.511 (0.248)	0.511 (0.248)	0.323 (0.389)

**Table 2:** Comparison of model performance using K-L based model selection criteria for SSVS example. The first row indicates the independent variables (x) to be excluded in each model. The mid rule separates the models most frequently appeared using SSVS method (above) vs. the models with lower PAIC (below).

Exclusion	SSVS	LOO-CV	KCV	${\mathrm{PL}}_{p_{\mathrm{opt}}^{e}}$	BPIC	PAIC
4, 5	827	2603.85	2580.74	2527.32	2528.89	2529.60
2, 4, 5	627	2572.98	2564.92	2544.77	2533.90	2534.44
3, 4, 5, 11	595	2583.63	2572.59	2545.23	2539.79	2540.20
3, 4, 5	486	2593.10	2579.97	2567.85	2541.75	2542.32
3, 4	456	2590.36	2571.76	2538.80	2533.37	2533.97
4, 5, 11	390	2589.76	2573.04	**2526.77**	**2527.94**	**2528.58**
2, 3, 4, 5	315	2576.66	2577.17	2561.57	2553.29	2553.77
3, 4, 11	245	2579.53	2566.28	2565.22	2532.87	2533.42
2, 4, 5, 11	209	**2564.67**	**2559.36**	2540.41	2533.60	2534.03
2, 4	209	2741.46	2741.17	2737.46	2740.42	2740.51
5, 10, 12	n/a	2602.23	2572.86	**2519.41**	2525.07	2525.61
4, 12	n/a	2596.51	2570.94	2520.52	2524.31	2524.94
5, 12	n/a	2595.86	2570.32	2520.51	**2524.19**	**2524.90**
4, 5, 12	n/a	2596.67	2574.73	2525.65	2526.19	2526.86
4, 10, 12	n/a	2603.05	2573.80	2520.62	2525.17	2525.70
4, 5, 10, 12	n/a	2603.51	2577.86	2526.53	2527.06	2527.56

## Data Availability

Not applicable.

## Abbreviations

The following abbreviations are used in this manuscript:

AIC	Akaike information criterion
BPIC	Bayesian predictive information criterion
DIC	Deviance information criterion
K-L	Kullback–Leibler
PAIC	Posterior averaging information criterion
WAIC	Watanabe–Akaike information criterion

## Appendix A. Supplementary Materials for Proof of Theorem 1

### Appendix A.1. Some Important Notations

By the law of large numbers we have $\frac{1}{n}\log\left\{L\left(\theta |y\right)\pi \left(\theta \right)\right\}\to E_{\tilde{y}}\left[\log\left\{g\left(\tilde{y}|\theta \right)\pi_{0}\left(\theta \right)\right\}\right]$ as *n* tends to infinity. Denote $\theta_{0}$, $\hat{\theta }$ the expected and empirical posterior mode of the log unnormalized posterior density log{L(θ|y)π(θ)}, i.e.,

$$\begin{array}{ccc} \theta_{0} & = & \arg\max_{\theta }E_{\tilde{y}}\left[\log\left\{g\left(\tilde{y}|\theta \right)\pi_{0}\left(\theta \right)\right\}\right]\\ \hat{\theta } & = & \arg\max_{\theta }\frac{1}{n}\log\left\{L\left(\theta |y\right)\pi \left(\theta \right)\right\}, \end{array}$$

and let I(θ) and J(θ) denote the Bayesian Hessian matrix and Bayesian Fisher information matrix

$$I\left(\theta \right)=E_{\tilde{y}}\left(\frac{\partial \log\{g\left(\tilde{y}|\theta \right)\pi_{0}\left(\theta \right)\}}{\partial \theta }\frac{\partial \log\{g\left(\tilde{y}|\theta \right)\pi_{0}\left(\theta \right)\}}{\partial \theta^{\prime }}\right)$$

and

$$J\left(\theta \right)=-E_{\tilde{y}}\left(\frac{\partial^{2}\log\left\{g\left(\tilde{y}|\theta \right)\pi_{0}\left(\theta \right)\right\}}{\partial \theta \partial \theta^{\prime }}\right).$$

### Appendix A.2. Proof of Lemmas

We start with a few lemmas to support the proofs of Theorem 1.

**Lemma A1.** 
*Under the same regularity conditions of Theorem 1, $\sqrt{n}\left(\hat{\theta }-\theta_{0}\right)$ is asymptotically distributed as $N(0,J_{n}^{-1}\left(\theta_{0}\right)I\left(\theta_{0}\right)J_{n}^{-1}\left(\theta_{0}\right))$.*

**Proof.** Consider the Taylor expansion of $\frac{\partial \log\{L(\theta |y)\pi (\theta )\}}{\partial \theta }{|}_{\theta =\hat{\theta }}$ at $\theta_{0}$,

$$\begin{array}{ccc} \frac{\partial \log\{L(\theta |y)\pi (\theta )\}}{\partial \theta }{|}_{\theta =\hat{\theta }} & ≃ & \frac{\partial \log\{L(\theta |y)\pi (\theta )\}}{\partial \theta }{{|}_{\theta =\theta_{0}}+\frac{\partial^{2}\log\left\{L\left(\theta |y\right)\pi \left(\theta \right)\right\}}{\partial \theta \partial \theta^{\prime }}|}_{\theta =\theta_{0}}\left(\hat{\theta }-\theta_{0}\right)\\ & = & \frac{\partial \log\{L(\theta |y)\pi (\theta )\}}{\partial \theta }{|}_{\theta =\theta_{0}}-nJ_{n}\left(\theta_{0}\right)\left(\hat{\theta }-\theta_{0}\right). \end{array}$$

Note that $\hat{\theta }$ is the mode of log{L(y|θ)π(θ)} and satisfies $\frac{\partial \log\{L(y|\theta )\pi (\theta )\}}{\partial \theta }{|}_{\theta =\hat{\theta }}=0$. Plug it into the above equation, we have

(A1) $$nJ_{n}\left(\theta_{0}\right)\left(\hat{\theta }-\theta_{0}\right)≃\frac{\partial \log\{L(\theta |y)\pi (\theta )\}}{\partial \theta }{|}_{\theta =\theta_{0}}.$$

From the central limit theorem, the right-hand-side (RHS) of Equation (A1) is approximately distributed as $N(0,nI\left(\theta_{0}\right))$ when $E_{y}\frac{\partial \log\{L(\theta |y)\pi (\theta )\}}{\partial \theta }{|}_{\theta =\theta_{0}}\to 0$. Therefore,

$$\sqrt{n}\left(\hat{\theta }-\theta_{0}\right)∼N\left(0,J_{n}^{-1}\left(\theta_{0}\right)I\left(\theta_{0}\right)J_{n}^{-1}\left(\theta_{0}\right)\right).$$

□

**Lemma A2.** 
*Under the same regularity conditions of Theorem 1, $\sqrt{n}\left(\theta -\hat{\theta }\right)∼N\left(0,J_{n}^{-1}\left(\hat{\theta }\right)\right)$.*

**Proof.** Taylor-expand the logarithm of L(θ|y)π(θ) around the posterior mode $\hat{\theta }$

(A2) $$\log L\left(\theta |y\right)\pi \left(\theta \right)=\log L\left(\hat{\theta }|y\right)\pi \left(\hat{\theta }\right)-\frac{1}{2}{\left(\theta -\hat{\theta }\right)}^{\prime }\frac{1}{n}J_{n}^{-1}\left(\hat{\theta }\right)\left(\theta -\hat{\theta }\right)+o_{p}\left(n^{-1}\right)$$

where $J_{n}\left(\hat{\theta }\right)=-\frac{1}{n}\frac{\partial^{2}\log\left\{L\left(\theta |y\right)\pi \left(\theta \right)\right\}}{\partial \theta \partial \theta^{\prime }}{|}_{\theta =\hat{\theta }}$. Consider the RHS of Equation (A2) as a function of θ: the first term is a constant, whereas the second term is proportional to the logarithm of a normal density. It yields the approximation of the posterior distribution for θ:

$$p\left(\theta |y\right)\approx N(\hat{\theta },\frac{1}{n}J_{n}^{-1}\left(\hat{\theta }\right)),$$

which completes the proof. Alternatively, though less intuitive, this lemma can also be proved by applying the Berstein–Von Mises theorem. □

**Lemma A3.** 
*Under the same regularity conditions of Theorem 1, $E_{\theta |y}\left(\theta_{0}-\hat{\theta }\right){\left(\hat{\theta }-\theta \right)}^{\prime }=o_{p}\left(n^{-1}\right)$.*

**Proof.** First we have

$$\frac{\partial \log\{L(\theta |y)\pi (\theta )\}}{\partial \theta }=\frac{\partial \log\{L(\theta |y)\pi (\theta )\}}{\partial \theta }{|}_{\theta =\hat{\theta }}-nJ_{n}\left(\hat{\theta }\right)\left(\theta -\hat{\theta }\right)+O_{p}\left(1\right).$$

Since $\hat{\theta }$ is the mode of log{L(θ|y)π(θ)}, it satisfies $\frac{\partial \log\{L(\theta |y)\pi (\theta )\}}{\partial \theta }{|}_{\theta =\hat{\theta }}=0$. Therefore, $\left(\hat{\theta }-\theta \right)=n^{-1}J_{n}^{-1}\left(\hat{\theta }\right)\frac{\partial \log\{L(\theta |y)\pi (\theta )\}}{\partial \theta }+O_{p}\left(n^{-1}\right)$. Note that

$$\begin{array}{ccc} E_{\theta |y}\frac{\partial \log\{L(\theta |y)\pi (\theta )\}}{\partial \theta } & = & \int \frac{\partial \log\{L(\theta |y)\pi (\theta )\}}{\partial \theta }\frac{L(\theta |y)\pi (\theta )}{p(y)}d\theta \\ & = & \int \frac{1}{L(\theta |y)\pi (\theta )}\frac{\partial \{L(\theta |y)\pi (\theta )\}}{\partial \theta }\frac{L(\theta |y)\pi (\theta )}{p(y)}d\theta \\ & = & \frac{1}{p(y)}\int \frac{\partial \{L(\theta |y)\pi (\theta )\}}{\partial \theta }d\theta \\ & = & \frac{1}{p(y)}\frac{\partial }{\partial \theta }\int L\left(\theta |y\right)\pi \left(\theta \right)d\theta =\frac{\partial }{\partial \theta }1=0. \end{array}$$

Because of assumption (C1), the equation holds when we change the order of the integral and derivative. Therefore,

$$E_{\theta |y}\left(\hat{\theta }-\theta \right)=n^{-1}J_{n}^{-1}\left(\hat{\theta }\right)E_{\theta |y}\frac{\partial \log\{L(\theta |y)\pi (\theta )\}}{\partial \theta }+O_{p}\left(n^{-1}\right)=O_{p}\left(n^{-1}\right).$$

Together with $\theta_{0}-\hat{\theta }=$$O_{p}\left(n^{-1/2}\right)$ derived from Lemma A1, we complete the proof. □

**Lemma A4.** 
*Under the same regularity conditions of Theorem 1, $E_{\theta |y}\left(\theta_{0}-\theta \right){\left(\theta_{0}-\theta \right)}^{\prime }=\frac{1}{n}J_{n}^{-1}\left(\hat{\theta }\right)+\frac{1}{n}J_{n}^{-1}\left(\theta_{0}\right)I\left(\theta_{0}\right)J_{n}^{-1}\left(\theta_{0}\right)+o_{p}\left(n^{-1}\right).$*

**Proof.** $E_{\theta |y}\left(\theta_{0}-\theta \right){\left(\theta_{0}-\theta \right)}^{\prime }$ can be rewritten as $\left(\theta_{0}-\hat{\theta }\right){\left(\theta_{0}-\hat{\theta }\right)}^{\prime }+E_{\theta |y}\left(\hat{\theta }-\theta \right){\left(\hat{\theta }-\theta \right)}^{\prime }+2E_{\theta |y}\left(\theta_{0}-\hat{\theta }\right)\left(\hat{\theta }-\theta \right)$. Applying Lemmas A1–A3, we complete the proof. □

**Lemma A5.** 
*Under the same regularity conditions of Theorem 1,*

$$\begin{array}{ccc} E_{\theta |y}\frac{1}{n}\log\left\{L\left(y|\theta \right)\pi \left(\theta \right)\right\} & ≃ & \frac{1}{n}\log\left\{L\left(\theta_{0}|y\right)\pi \left(\theta_{0}\right)\right\}\\ & & +\frac{1}{2n}\left(tr\left\{J_{n}^{-1}\left(\theta_{0}\right)I\left(\theta_{0}\right)\right\}-tr\left\{J_{n}^{-1}\left(\hat{\theta }\right)J_{n}\left(\theta_{0}\right)\right\}\right)+O_{p}\left(n^{-1}\right). \end{array}$$

**Proof.** The posterior mean of the log joint density distribution of (y,θ) can be Taylor-expanded around $\theta_{0}$ as

(A3) $$\begin{array}{ccc} E_{\theta |y}\frac{1}{n}\log\left\{L\left(\theta |y\right)\pi \left(\theta \right)\right\} & = & \frac{1}{n}\log\left\{L\left(\theta_{0}|y\right)\pi \left(\theta_{0}\right)\right\}+E_{\theta |y}{\left(\theta -\theta_{0}\right)}^{\prime }\frac{1}{n}\frac{\partial \log\{L(\theta |y)\pi (\theta )\}}{\partial \theta }{|}_{\theta =\theta_{0}}\\ & & +\frac{1}{2}E_{\theta |y}{\left(\theta -\theta_{0}\right)}^{\prime }\frac{1}{n}\frac{\partial^{2}\log\left\{L\left(\theta |y\right)\pi \left(\theta \right)\right\}}{\partial \theta \partial \theta^{\prime }}{|}_{\theta =\theta_{0}}\left(\theta -\theta_{0}\right)+o_{p}\left(n^{-1}\right)\\ & = & \frac{1}{n}\log\left\{L\left(\theta_{0}|y\right)\pi \left(\theta_{0}\right)\right\}+E_{\theta |y}{\left(\theta -\theta_{0}\right)}^{\prime }\frac{1}{n}\frac{\partial \log\{L(\theta |y)\pi (\theta )\}}{\partial \theta }{|}_{\theta =\theta_{0}}\\ & & -\frac{1}{2}E_{\theta |y}{\left(\theta -\theta_{0}\right)}^{\prime }J_{n}\left(\theta_{0}\right)\left(\theta -\theta_{0}\right)+o_{p}\left(n^{-1}\right). \end{array}$$

Expand $\frac{\partial \log\{L(\theta |y)\pi (\theta )\}}{\partial \theta }{|}_{\theta =\hat{\theta }}$ around $\theta_{0}$ to the first order, we obtain

(A4) $$\frac{\partial \log\{L(\theta |y)\pi (\theta )\}}{\partial \theta }{{|}_{\theta =\hat{\theta }}=\frac{\partial \log\{L(\theta |y)\pi (\theta )\}}{\partial \theta }|}_{\theta =\theta_{0}}-nJ_{n}\left(\theta_{0}\right)\left(\hat{\theta }-\theta_{0}\right)+O_{p}\left(n^{-1}\right).$$

Because the posterior mode $\hat{\theta }$ is the solution of $\frac{\partial \log\{L(\theta |y)\pi (\theta )\}}{\partial \theta }=0$, Equation (A4) can be re-written as

$$\frac{1}{n}\frac{\partial \log\{L(\theta |y)\pi (\theta )\}}{\partial \theta }{|}_{\theta =\theta_{0}}=J_{n}\left(\theta_{0}\right)\left(\hat{\theta }-\theta_{0}\right)+O_{p}\left(n^{-1}\right).$$

Substituting it into the second term of (A3), the expansion of $E_{\theta |y}\frac{1}{n}\log\left\{L\left(\theta |y\right)\pi \left(\theta \right)\right\}$ becomes:

$$\begin{array}{ccc} E_{\theta |y}\frac{1}{n}\log\left\{L\left(\theta |y\right)\pi \left(\theta \right)\right\} & = & \frac{1}{n}\log\left\{L\left(\theta_{0}|y\right)\pi \left(\theta_{0}\right)\right\}+E_{\theta |y}{\left(\theta -\theta_{0}\right)}^{\prime }J_{n}\left(\theta_{0}\right)\left(\hat{\theta }-\theta_{0}\right)\\ & & -\frac{1}{2}E_{y}E_{\theta |y}{\left(\theta -\theta_{0}\right)}^{\prime }J_{n}\left(\theta_{0}\right)\left(\theta -\theta_{0}\right)+o_{p}\left(n^{-1}\right)\\ & = & \frac{1}{n}\log\left\{L\left(\theta_{0}|y\right)\pi \left(\theta_{0}\right)\right\}+tr\left\{E_{\theta |y}\left[\left(\hat{\theta }-\theta_{0}\right){\left(\theta -\theta_{0}\right)}^{\prime }\right]J_{n}\left(\theta_{0}\right)\right\}\\ & & -\frac{1}{2}tr\left\{E_{\theta |y}\left[\left(\theta -\theta_{0}\right){\left(\theta -\theta_{0}\right)}^{\prime }\right]J_{n}\left(\theta_{0}\right)\right\}+o_{p}\left(n^{-1}\right)\\ & = & \frac{1}{n}\log\left\{L\left(\theta_{0}|y\right)\pi \left(\theta_{0}\right)\right\}+tr\left\{E_{\theta |y}\left[\left(\theta -\theta_{0}\right){\left(\hat{\theta }-\theta_{0}\right)}^{\prime }\right]J_{n}\left(\theta_{0}\right)\right\}\\ & & -\frac{1}{2}tr\left\{\frac{1}{n}\left[J_{n}^{-1}\left(\hat{\theta }\right)+J_{n}^{-1}\left(\theta_{0}\right)I\left(\theta_{0}\right)J_{n}^{-1}\left(\theta_{0}\right)\right]J_{n}\left(\theta_{0}\right)\right\}+o_{p}\left(n^{-1}\right) \end{array}$$

where in the last line we replace $E_{\theta |y}\left[\left(\theta -\theta_{0}\right){\left(\theta -\theta_{0}\right)}^{\prime }\right]$ with the result of Lemma A4. $E_{\theta |y}\left[\left(\theta -\theta_{0}\right){\left(\hat{\theta }-\theta_{0}\right)}^{\prime }\right]$ in the second term of the expansion can be rewritten as $E_{\theta |y}\left[\left(\hat{\theta }-\theta_{0}\right){\left(\hat{\theta }-\theta_{0}\right)}^{\prime }\right]+E_{\theta |y}\left[\left(\theta -\hat{\theta }\right){\left(\hat{\theta }-\theta_{0}\right)}^{\prime }\right]$, where the former term is asymptotically equal to $\frac{1}{n}J_{n}^{-1}\left(\theta_{0}\right)I\left(\theta_{0}\right)J_{n}^{-1}\left(\theta_{0}\right)$ by Lemma A1, and the latter is negligible with higher order $o_{p}\left(n^{-1}\right)$, as shown in Lemma A3. Therefore, the expansion can be finally simplified as

$$\begin{array}{ccc} E_{\theta |y}\frac{1}{n}\log\left\{L\left(y|\theta \right)\pi \left(\theta \right)\right\} & ≃ & \frac{1}{n}\log\left\{L\left(\theta_{0}|y\right)\pi \left(\theta_{0}\right)\right\}\\ & & +\frac{1}{2n}\left(tr\left\{J_{n}^{-1}\left(\theta_{0}\right)I\left(\theta_{0}\right)\right\}-tr\left\{J_{n}^{-1}\left(\hat{\theta }\right)J_{n}\left(\theta_{0}\right)\right\}\right)+O_{p}\left(n^{-1}\right). \end{array}$$

□

## Appendix B. Supplementary Materials for Derivation of Equation (3)

We start from Equation (2), which rewrites Equation (5) in Ando [7].

$$\begin{array}{ccc} \;{\hat{\eta }}^{BPIC} & = & \frac{1}{n}E_{\theta |y}\log L\left(\theta |y\right)-\frac{1}{n}[\;E_{\theta |y}\log\left\{\pi \left(\theta \right)L\left(\theta |y\right)\right\}-\log\left\{\pi \left(\hat{\theta }\right)L\left(\hat{\theta }|y\right)\right\}\\ & & +tr\left\{J_{n}^{-1}\left(\hat{\theta }\right)I_{n}\left(\hat{\theta }\right)\right\}+\frac{K}{2}]\\ & = & \frac{1}{n}E_{\theta |y}\log L\left(\theta |y\right)-\frac{1}{n}[\;E_{\theta |y}\log\pi \left(\theta \right)+E_{\theta |y}\log L\left(\theta |y\right)-\log\pi \left(\hat{\theta }\right)-\log L\left(\hat{\theta }|y\right)\\ & & +tr\left\{J_{n}^{-1}\left(\hat{\theta }\right)I_{n}\left(\hat{\theta }\right)\right\}+\frac{K}{2}]\\ & = & \frac{1}{n}E_{\theta |y}\log L\left(\theta |y\right)-\frac{1}{n}\;E_{\theta |y}\log\pi \left(\theta \right)-\frac{1}{n}E_{\theta |y}\log L\left(\theta |y\right)+\frac{1}{n}\log\pi \left(\hat{\theta }\right)+\frac{1}{n}\log L\left(\hat{\theta }|y\right)\\ & & -\frac{1}{n}tr\left\{J_{n}^{-1}\left(\hat{\theta }\right)I_{n}\left(\hat{\theta }\right)\right\}-\frac{K}{2n}\\ & = & \frac{1}{n}\log L\left(\hat{\theta }|y\right)-\frac{1}{n}\;E_{\theta |y}\log\pi \left(\theta \right)+\frac{1}{n}\log\pi \left(\hat{\theta }\right)-\frac{1}{n}tr\left\{J_{n}^{-1}\left(\hat{\theta }\right)I_{n}\left(\hat{\theta }\right)\right\}-\frac{K}{2n}\\ & = & \frac{1}{n}\log L\left(\hat{\theta }|y\right)-\frac{1}{n}\left[\;E_{\theta |y}\log\pi \left(\theta \right)-\log\pi \left(\hat{\theta }\right)+tr\left\{J_{n}^{-1}\left(\hat{\theta }\right)I_{n}\left(\hat{\theta }\right)\right\}+\frac{K}{2}\right]\\ & ≜ & \frac{1}{n}\log L\left(\hat{\theta }|y\right)-BC_{2}. \end{array}$$
